# Supplementary material for: Gemcitabine: An Alternative Treatment for Oxaliplatin-Resistant Colorectal Cancer
Source: Cancers (Basel). 2022 Nov 29;14(23):5894. doi: 10.3390/cancers14235894 (PMC9740936; doi:10.3390/cancers14235894)
Supplement: Supplementary file 1 [file cancers-14-05894-s001.zip › cancers-1978477-supplementary (3).pdf]

Drugs	Classification	Cancer	p38 action
Doxorubicin	Anthracycline antibiotic<br>Interspersing DNA<br>Topoisomerase II inhibitor	advanced colorectal, gastric,<br>pancreatic and lung cancer	resistance
Etoposide	Topoisomerase II inhibitor	ovarian, lung, testicular, leukemia<br>lymphoma and neuroblastoma	resistance
5-Fluorouracil	Antimetabolite<br>Pyrimidine analog	ovarian, colon, cervical, esophageal<br>cervical and pancreatic cancer	ambiguous
Gemcitabine	Antimetabolite<br>Pyrimidine analog	ovarian, breast, bladder,<br>and pancreatic cancer	ambiguous
Oxaliplatin	Platinum-based<br>antineoplastic	advanced colorectal, gastric,<br>pancreatic and lung cancer	ambiguous
Paclitaxel	Taxane<br>Spindle poison<br>Depolymeristion inhibitor	ovarian, breast, lung, cervical,<br>and pancreatic cancer	ambiguous
Vincristine	Vinca alkaloid<br>Spindle poison<br>Polymeristion inhibitor	acute lymphocytic leukemia<br>neuroblastoma<br>small cell lung cancer	ambiguous

Table S1: This table presents the drugs used in our project, their drug classification (classification), the cancers targeted (cancer) and p38 effect on treatment efficiency (p38 action).

MAPK signaling pathway			MAPK signaling pathway		
PTK/STK	Rank	Protein name	PTK/STK	Rank	Protein name
STK	93/158	AKT3	STK	6/158	MAPK13
STK	22/158	ARAF	STK	7/158	MAPK14
STK	21/158	BRAF	STK	8/158	MAPK3
STK	48/158	CHUK	STK	3/158	MAPK7
PTK	58/67	EGFR	STK	15/158	MAPK8
PTK	37/67	EPHA2	STK	11/158	MAPK9
PTK	15/67	ERBB2	STK	94/158	MAPKAPK2
PTK	26/67	ERBB3	STK	50/158	NLK
PTK	24/67	ERBB4	PTK	14/67	NTRK1
PTK	62/67	FGFR1	PTK	13/67	NTRK2
PTK	61/67	FGFR2	PTK	31/67	PDGFRA
PTK	65/67	FGFR3	PTK	60/67	PDGFRB
PTK	5/67	FGFR4	STK	67/158	PRKCG
PTK	67/67	FLT1	STK	25/158	RAF1
PTK	55/67	FLT3	STK	68/158	RPS6KA1
PTK	51/67	FLT4	STK	44/158	RPS6KA2
PTK	17/67	IGF1R	STK	62/158	RPS6KA3
STK	27/158	IKBKB	STK	92/158	RPS6KA5
PTK	3/67	INSR	STK	74/158	TAOK1
PTK	42/67	KDR	STK	73/158	TAOK2
PTK	1/67	KIT	STK	72/158	TAOK3
STK	155/158	MAP2K1			
STK	158/158	MAP2K2			
STK	53/158	MAP3K8			
STK	9/158	MAPK1			
STK	14/158	MAPK10			
STK	23/158	MAPK11			
STK	10/158	MAPK12			

Table S2: this table presents the kinases studied in the MAPK signaling pathway interactome, their status (serine (STK) or threonine/tyrosine kinase (PTK)), their rank in the PamGene analyses (Rank) and their name (protein name).

PI3K-Akt signaling pathway		
PTK/STK	Rank	Protein name
STK	93/158	AKT3
STK	5/158	CDK2
STK	58/158	CDK4
STK	42/158	CDK6
STK	48/158	CHUK
PTK	58/67	EGFR
PTK	37/67	EPHA2
PTK	15/67	ERBB2
PTK	26/67	ERBB3
PTK	24/67	ERBB4
PTK	62/67	FGFR1
PTK	61/67	FGFR2
PTK	65/67	FGFR3
PTK	5/67	FGFR4
PTK	67/67	FLT1
PTK	55/67	FLT3
PTK	51/67	FLT4
STK	36/158	GSK3B
PTK	17/67	IGF1R
STK	27/158	IKBKB
PTK	3/67	INSR
PTK	27/67	JAK2
PTK	42/67	KDR
PTK	1/67	KIT
STK	155/158	MAP2K1
STK	158/158	MAP2K2
STK	9/158	MAPK1
STK	8/158	MAPK3
STK	71/158	MTOR
PTK	14/67	NTRK1
PTK	13/67	NTRK2
PTK	31/67	PDGFRA
PTK	60/67	PDGFRB
STK	97/158	PRKAA1
PTK	8/67	PTK2
STK	25/158	RAF1
PTK	47/67	SYK

Table S3: this table presents the kinases studied in PI3K-Akt signaling pathway interactome, their status (serine (STK) or threonine/tyrosine kinase (PTK)), their rank in the PamGene analyses (Rank) and their name (protein name).

EGFR tyrosine kinase inhibitor resistance		
PTK/STK	Rank	Protein name
STK	93/158	AKT3
STK	22/158	ARAF
PTK	18/67	AXL
STK	21/158	BRAF
PTK	58/67	EGFR
PTK	15/67	ERBB2
PTK	26/67	ERBB3
PTK	61/67	FGFR2
PTK	65/67	FGFR3
STK	36/158	GSK3B
PTK	17/67	IGF1R
PTK	27/67	JAK2
PTK	42/67	KDR
STK	155/158	MAP2K1
STK	158/158	MAP2K2
STK	9/158	MAPK1
STK	8/158	MAPK3
STK	71/158	MTOR
PTK	31/67	PDGFRA
PTK	60/67	PDGFRB
STK	67/158	PRKCG
STK	25/158	RAF1
PTK	2/67	SRC

Table S4: this table presents the kinases studied in the EGFR tyrosine kinase inhibitor resistance interactome, their status (serine (STK) or threonine/tyrosine kinase (PTK)), their rank in the PamGene analyses (Rank) and their name (protein name).

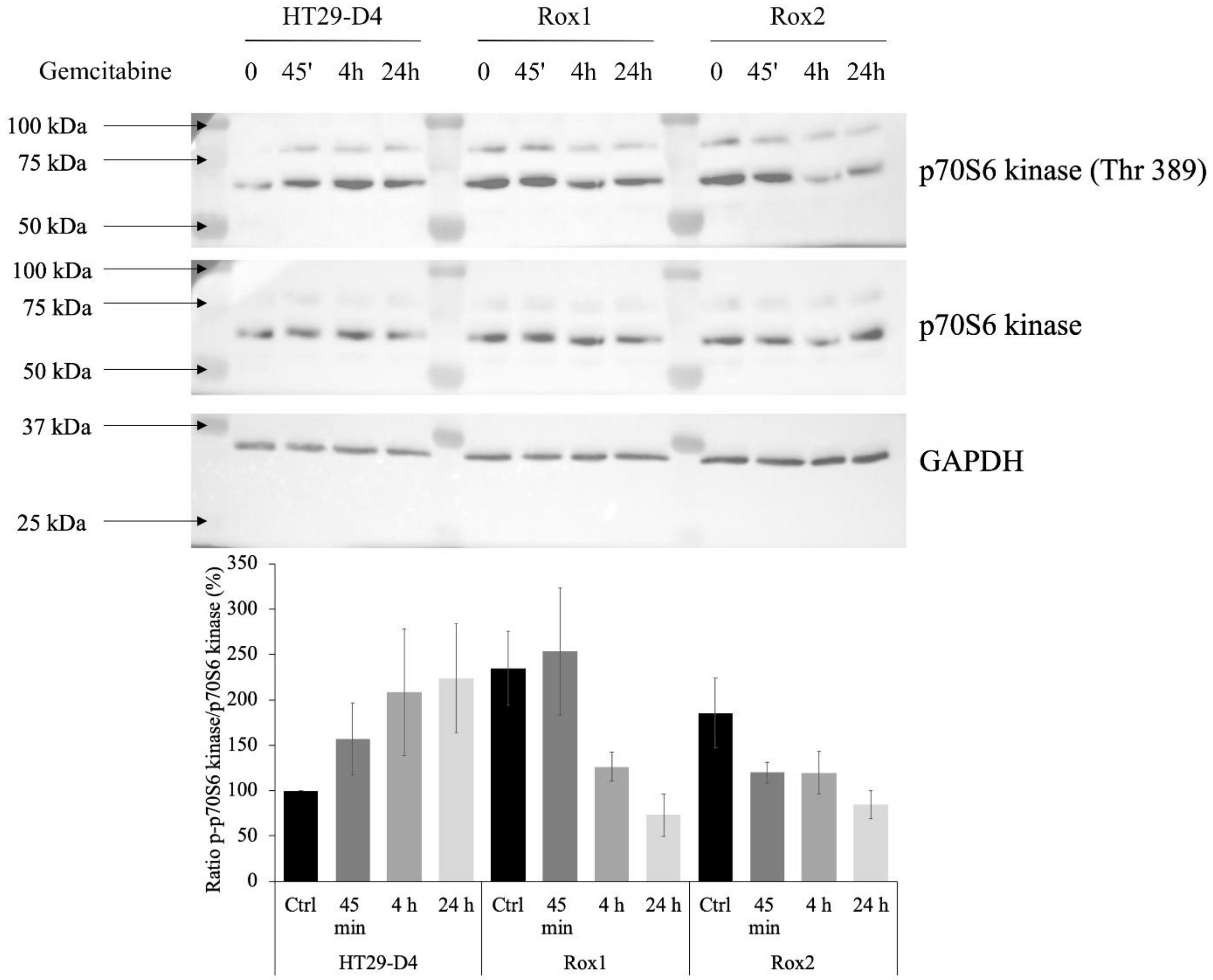

Figure S1: Effects of gemcitabine on the phosphorylation of p70S6 kinase. The HT29-D4, Rox1 and Rox2 cells were seeded in 6 well-plates. After 24 hours, the cells were treated with gemcitabine for 45 minutes, 4 hours and 24 hours. The cells were then lysed, and the extracted proteins were used to study the phosphorylation of p70S6 kinase (T389) by Western blot. Band intensities were quantified using ImageJ, normalized using GAPDH expression and expressed as ratios (p-p70S6 / p70S6, 100% for untreated HT29-D4).

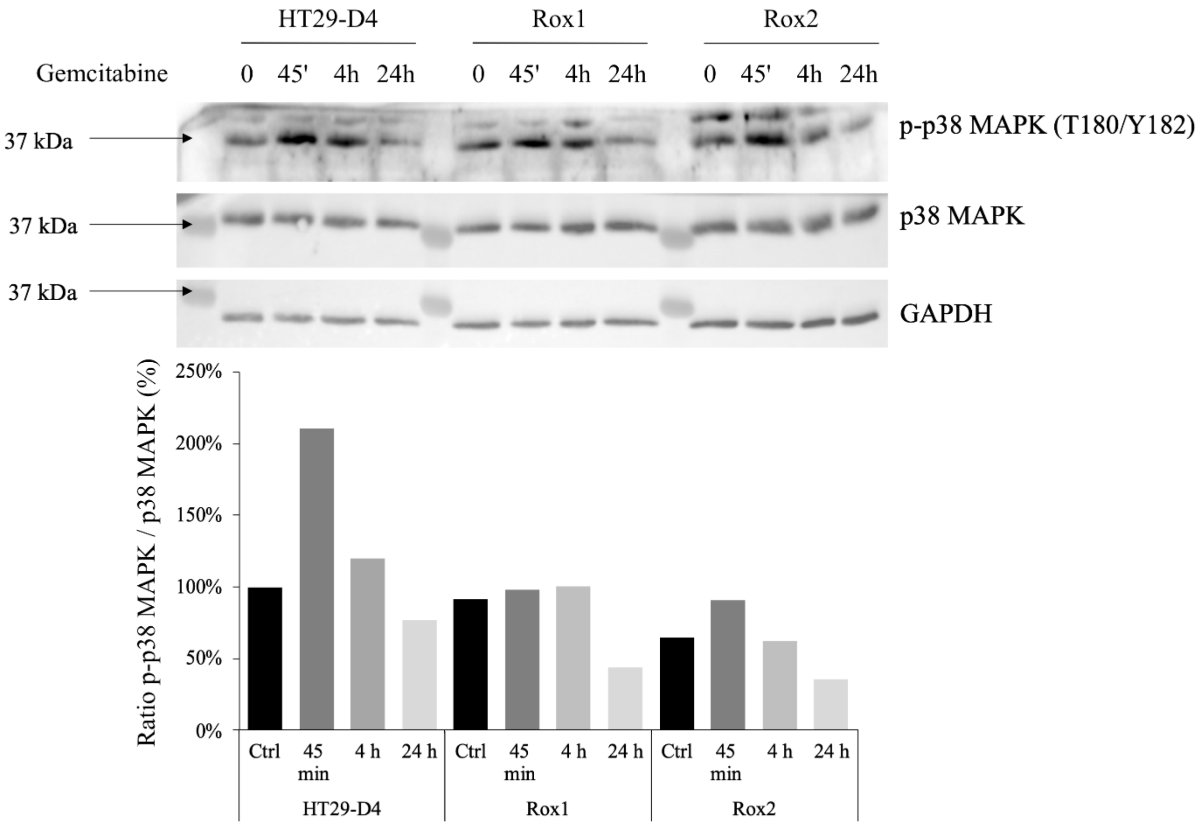

Figure S2: Effects of gemcitabine on p38 MAPK phosphorylation. The HT29-D4, Rox1 and Rox2 cells were seeded in 6 well-plates. After 24 hours, the cells were treated with gemcitabine for 45 minutes, 4 hours and 24 hours. The cells were then lysed, and the extracted proteins were used to study the phosphorylation of p38 MAPK (T180 / Y182) by Western blot. Band intensities were quantified using ImageJ, normalized using GAPDH expression and expressed as ratios (p-p38 / p38, 100% for untreated HT29-D4).
