# Supplementary material for: Gemcitabine: An Alternative Treatment for Oxaliplatin-Resistant Colorectal Cancer
Source: Cancers (Basel). 2022 Nov 29;14(23):5894. doi: 10.3390/cancers14235894 (PMC9740936; doi:10.3390/cancers14235894)

Immunoblot

Raw material

# Figure 3F : PARP & cleaved PARP

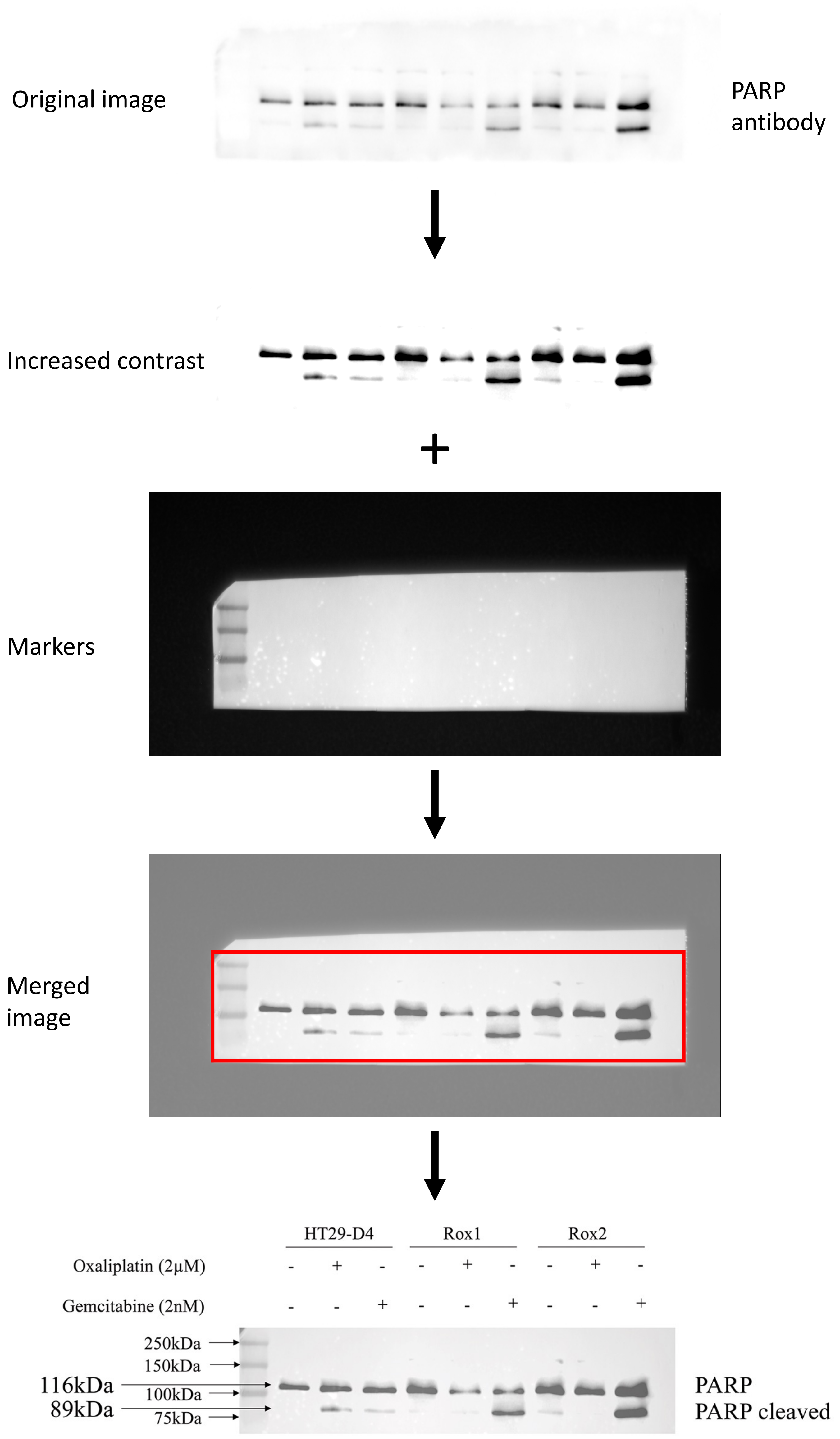

# Figure 3F : GAPDH

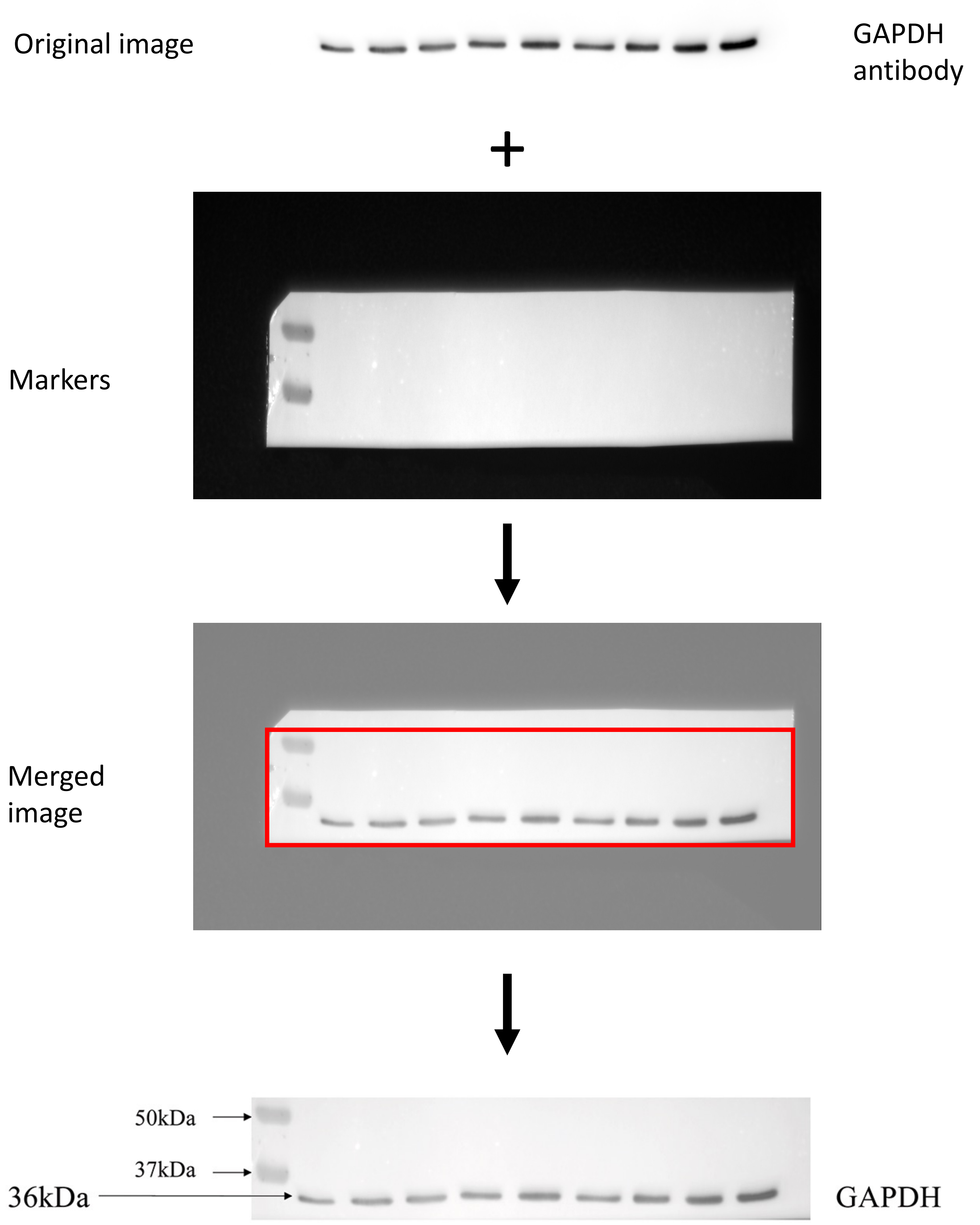

# Figure 3F : densitometric analysis

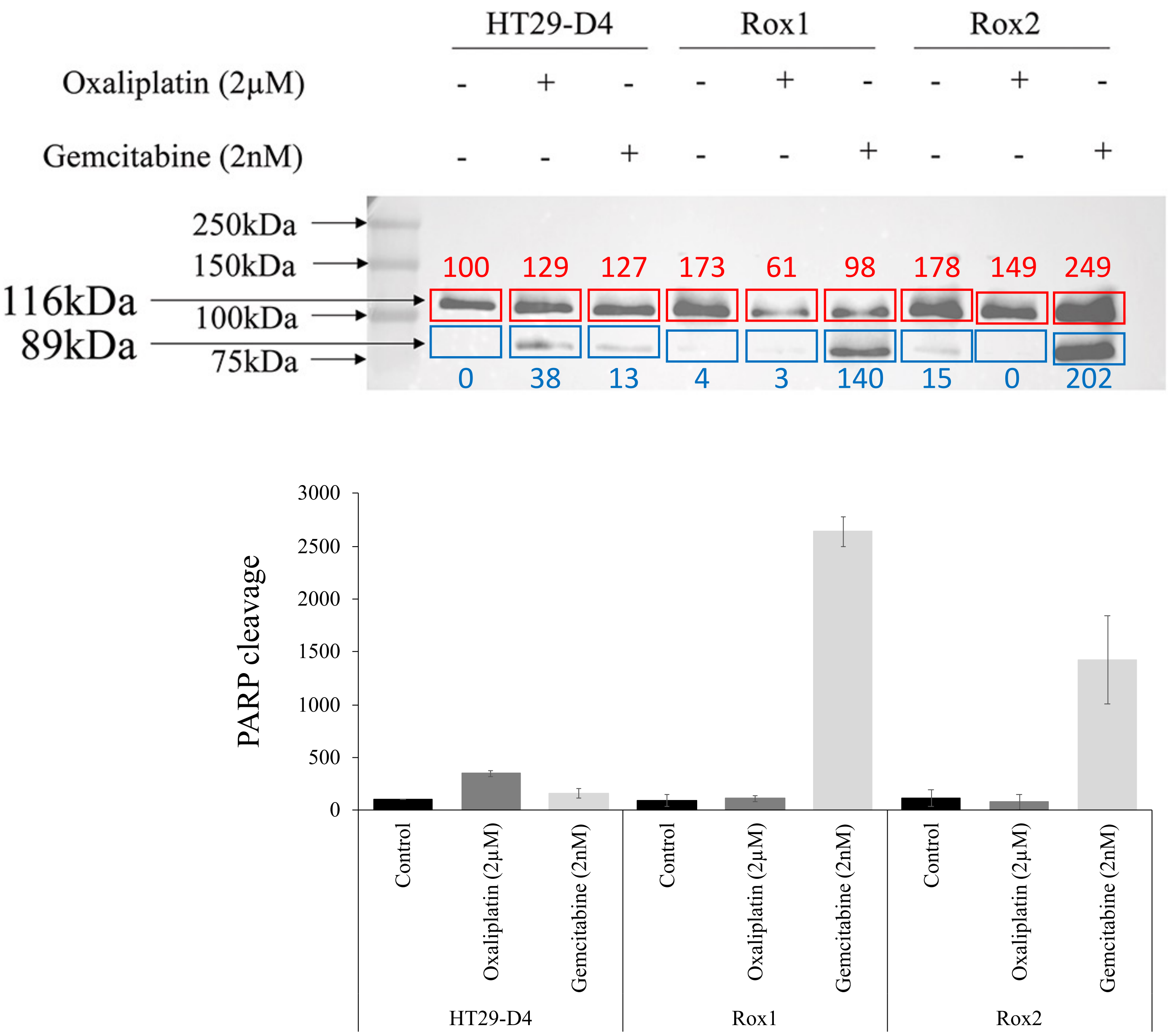

# Figure S1 : p70S6

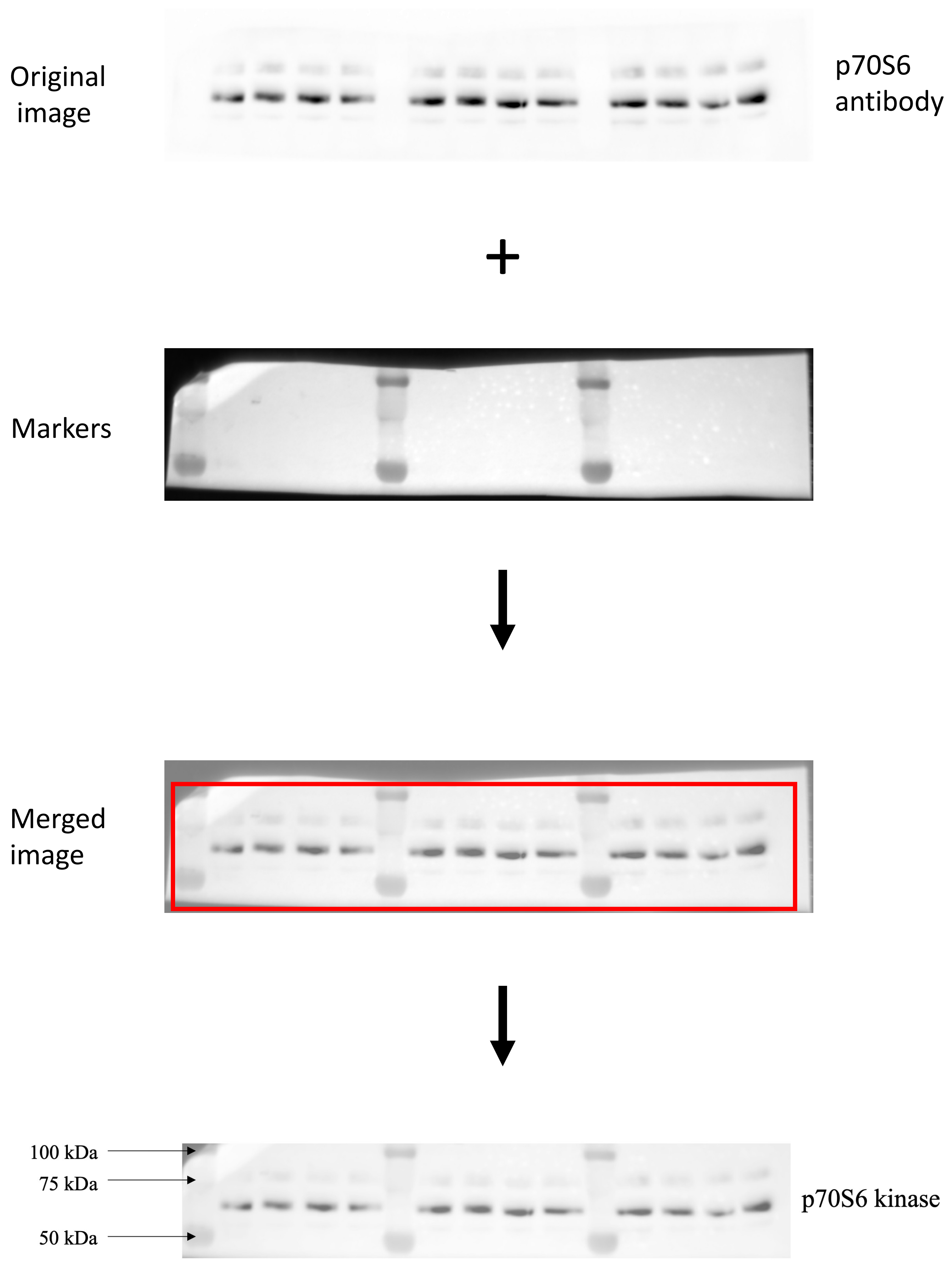

# Figure S1 : p-p70S6

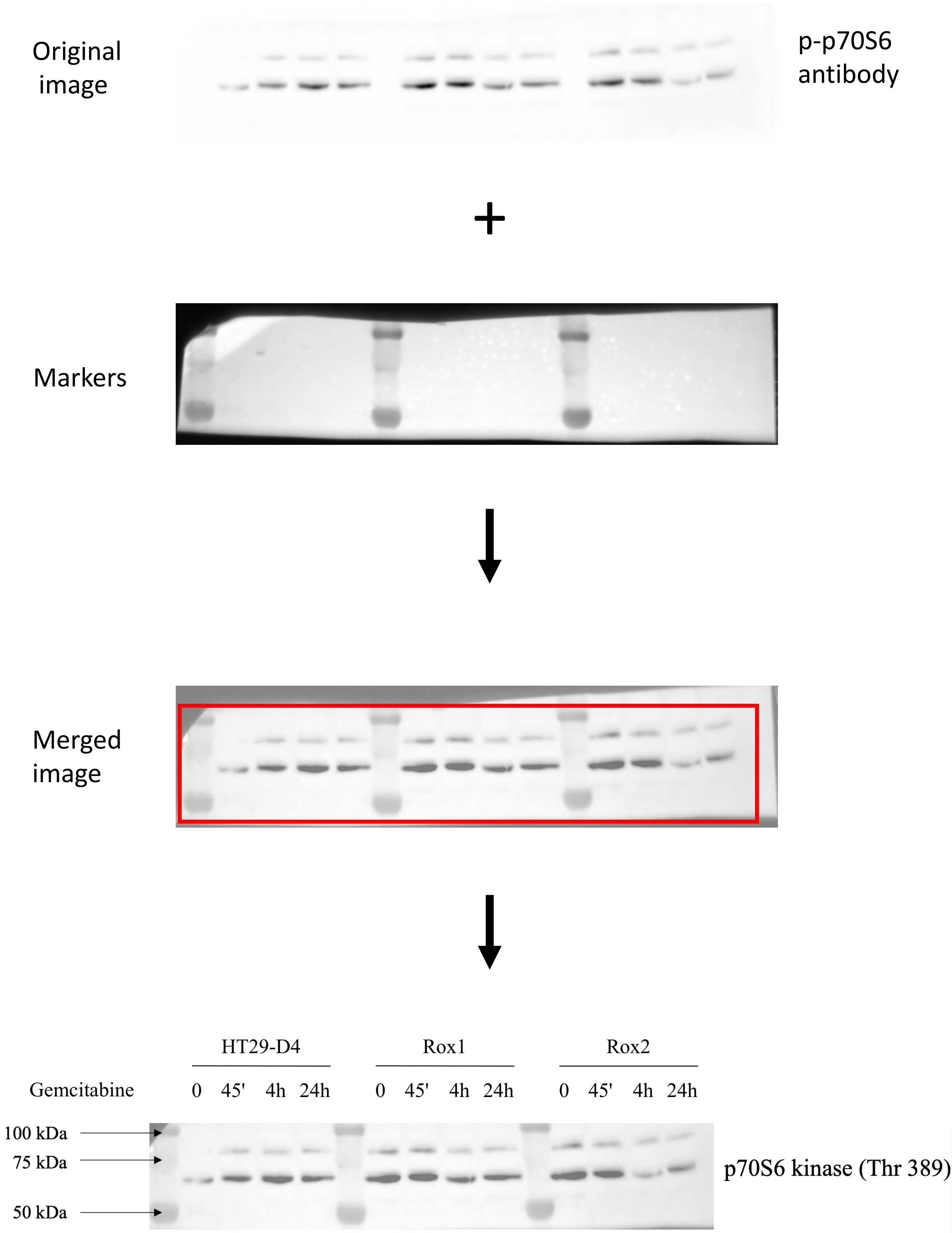

# Figure S1 : GAPDH

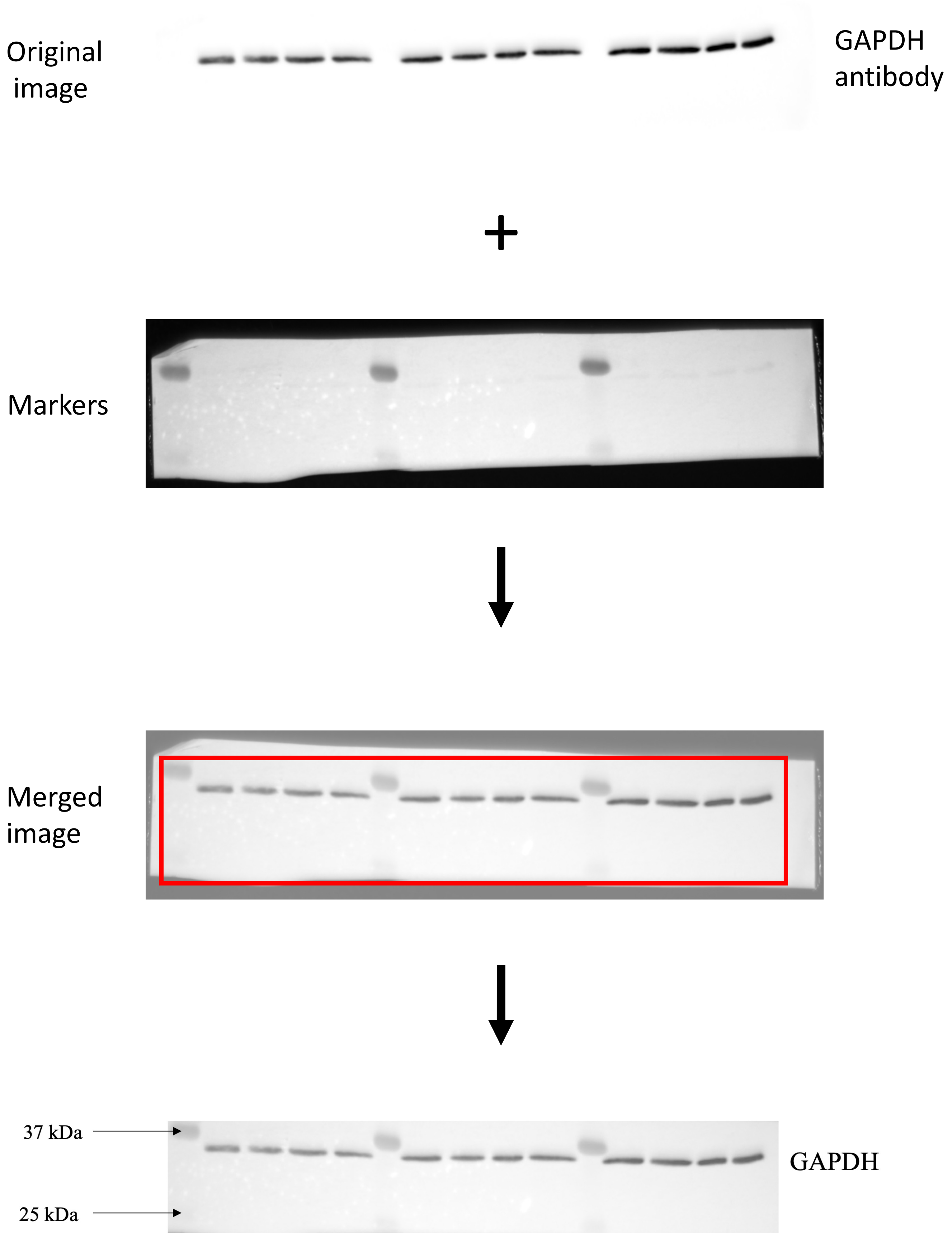

# Figure S2 : p38 MAPK

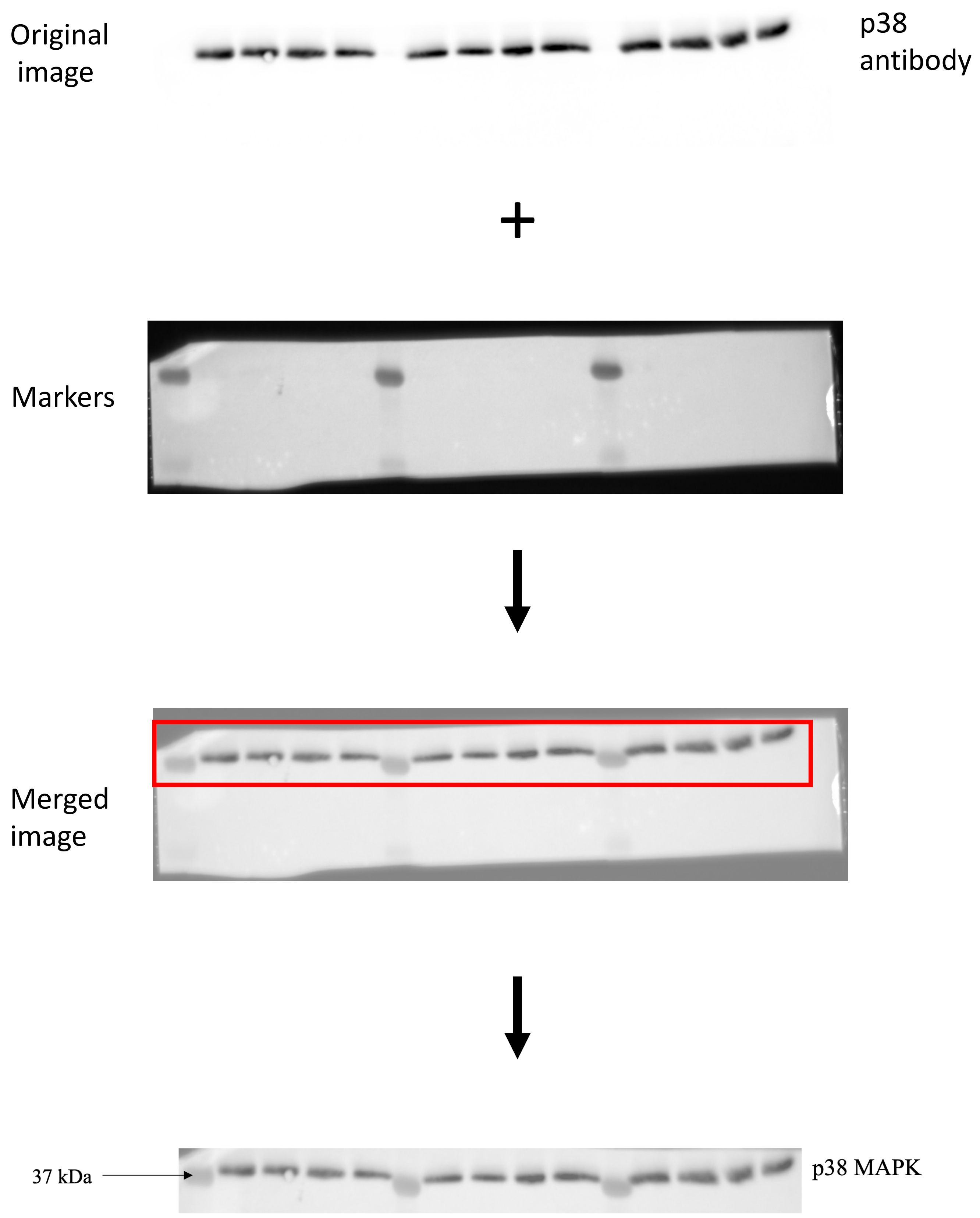

# Figure S2 : p-p38 MAPK

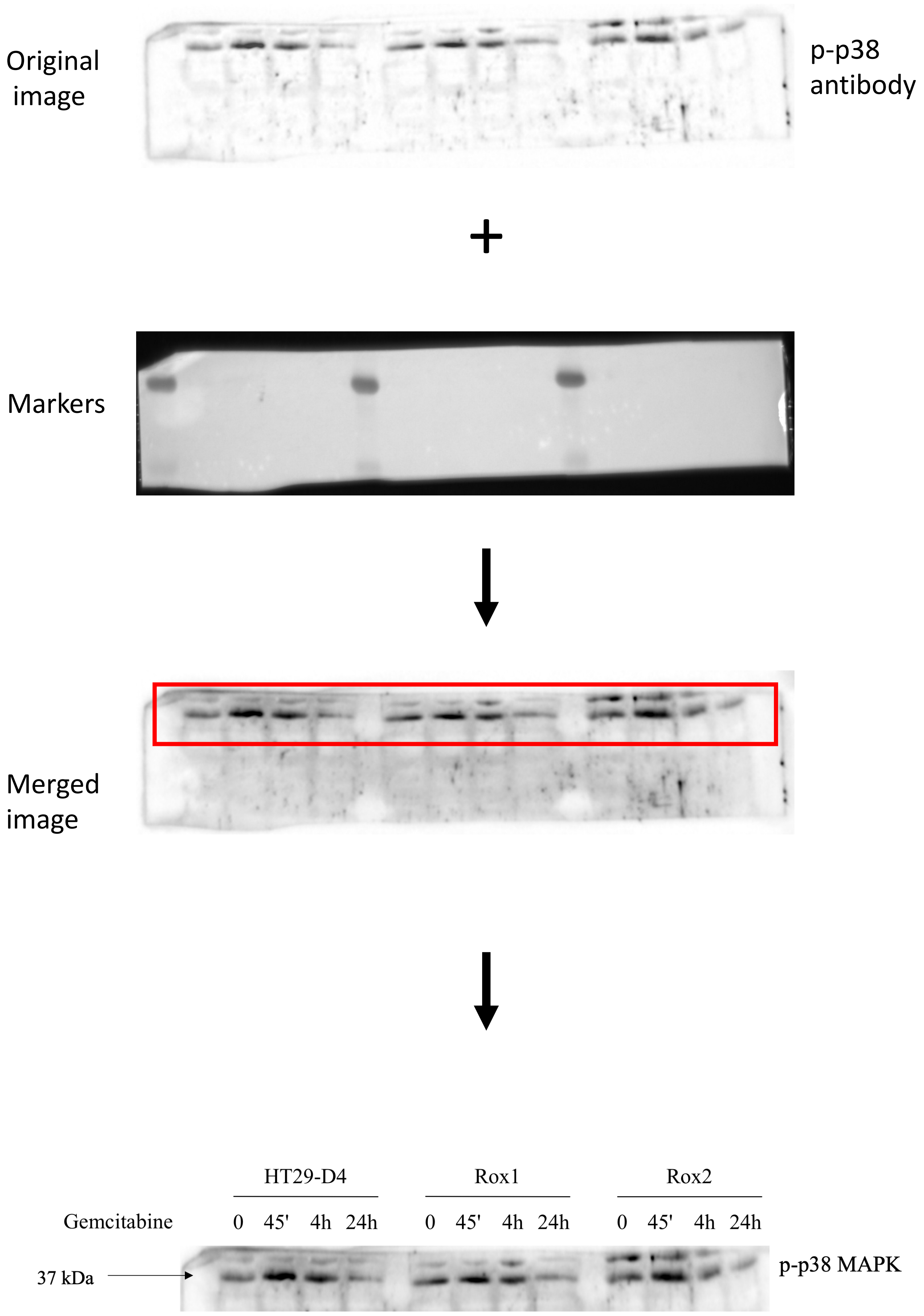

# Figure S2 : GAPDH

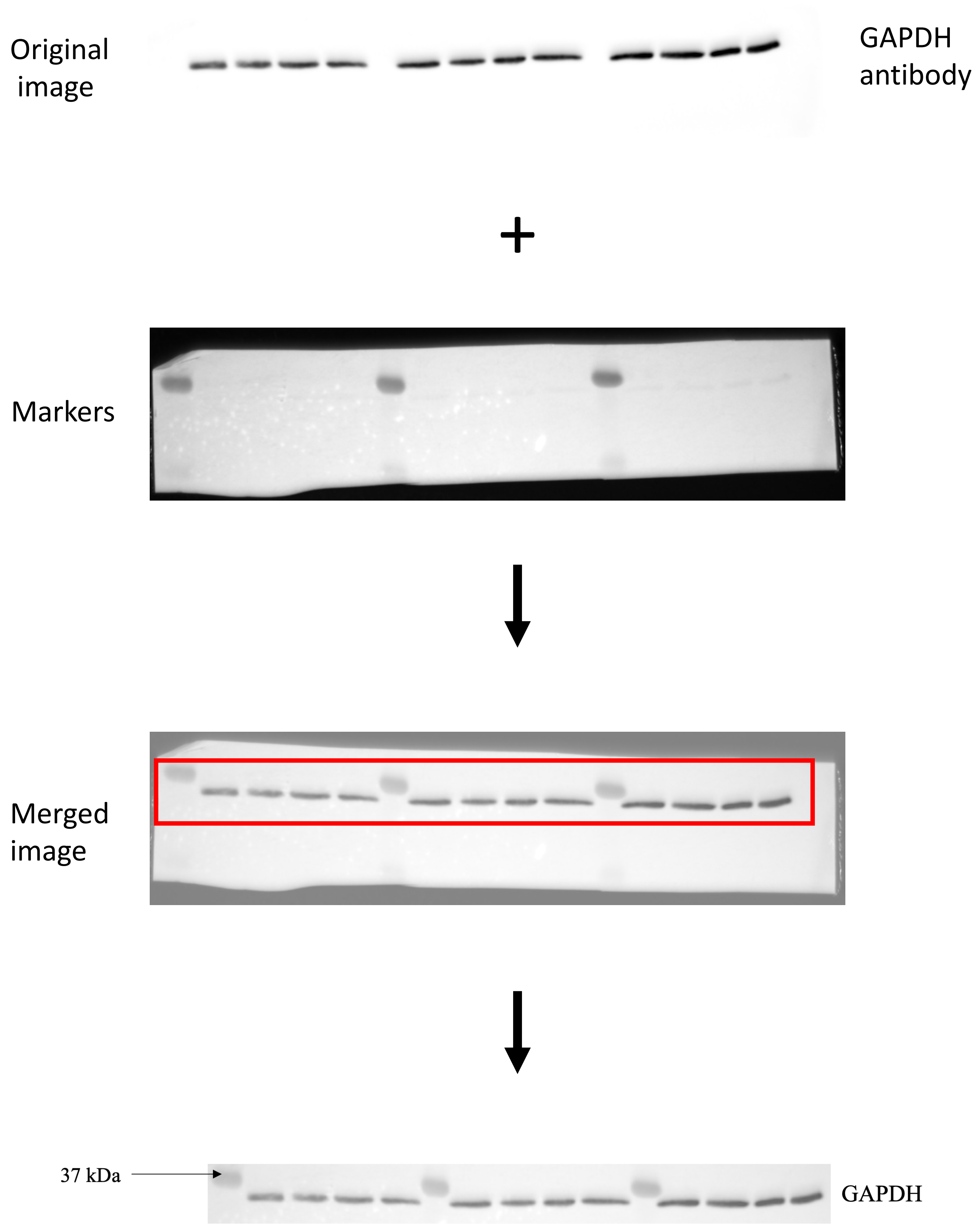

Supplement: Supplementary file 1 [file cancers-14-05894-s001.zip › Supplemental data original WB.pdf]
